# Effect of community mobilization on appropriate care seeking for pneumonia in Haripur, Pakistan

**DOI:** 10.7189/jogh.05.010405

**Published:** 2015-06

**Authors:** Salim Sadruddin, Ibad ul Haque Khan, Abdul Bari, Attaullah Khan, Ijaz Ahmad, Shamim A. Qazi

**Affiliations:** 1 Save the Children US, Fairfield, CT, USA; 2 Save the Children, Pakistan Program, Islamabad, Pakistan; 3 Independent consultant, formerly with Save the Children, Pakistan; 4 Directorate General Health Services, Peshawar, Khyber Pakhtunkhwa, Pakistan; 5 Health Sector Reform Unit, Department of Health, Peshawar, Khyber Pakhtunkhwa, Pakistan; 6 World Health Organization, Department of Maternal, Newborn, Child and Adolescent Health, Geneva, Switzerland

## Abstract

**Background:**

Appropriate and timely care seeking reduces mortality for childhood illnesses including pneumonia. Despite over 90 000 Lady Health Workers (LHWs) deployed in Pakistan, whose tasks included management of pneumonia, only 16% of care takers sought care from them for respiratory infections. As part of a community case management trial for childhood pneumonia, community mobilization interventions were implemented to improve care seeking from LHWs in Haripur district, Pakistan. The objective of the study was to increase the number of children receiving treatment for pneumonia and severe pneumonia by Lady Health Workers (LHWs) through community mobilization approaches for prompt recognition and care seeking in 2 to 59 month–old children.

**Methods:**

To assess pneumonia care seeking practices, pre and post–intervention household surveys were conducted in 28 target Union Councils. Formative research to improve existing LHW training materials, job aids and other materials was carried out. Advocacy events were organized, LHWs and male health promoters were trained in community mobilization, non–functional women and male health committees were revitalized and LHWs and male health promoters conducted community awareness sessions.

**Results:**

The community mobilization interventions were implemented from April 2008 – December 2009. Project and LHW program staff organized 113 sensitization meetings for opinion leaders, which were attended by 2262 males and 3288 females. The 511 trained LHWs organized 6132 community awareness sessions attended by 50 056 women and 511 male promoters conducted 523 sessions attended by 7845 males. In one year period, the number of LHWs treating pneumonia increased from 11 in April 2008 to 505 in March 2009. The care seeking from LHWs for suspected pneumonia increased from 0.7% in pre–intervention survey to 49.2% in post–intervention survey.

**Conclusion:**

The increase in care seeking from LHWs benefited the community through bringing inexpensive appropriate care closer to home and reducing burden on overstretched health facilities. The community mobilization interventions led to improvements in appropriate care seeking that would not have been achievable just by strengthening pneumonia case management skills of LHWs. In addition to strengthening skills, community mobilization and behavior change activities should also be included in community case management programmes.

Over 120 million pneumonia cases occur annually resulting in an estimated one million deaths in children under five years of age [1–3]. Over 80% pneumonia deaths occur at home [4]. Pakistan is among the top five countries that contribute to majority of these pneumonia episodes and deaths globally [5].

In children, a non–fatal disease can progress within 2–3 days to a fatal outcome if appropriate care is not provided in time [6–9]. Where access to health care is low, WHO and UNICEF recommend that trained community health workers (CHWs) treat pneumonia with oral antibiotics [10]. This requires deployment of trained CHW in the community, household recognition of pneumonia and prompt care seeking. Interventions promoting care seeking improve mortality outcomes, but timely care seeking from an appropriate care provider is essential [11,12].

Recognition of a pneumonia episode by the care takers is low in developing countries including Pakistan [13]. Sometimes, they may recognize the symptoms, but delay care seeking [14]. Appropriate care seeking from health workers trained in standard pneumonia case management is low, particularly in rural and poorer communities [5]. A systematic review of studies from developing countries, including Pakistan found that care seeking from available CHWs for pneumonia was only 4.2% [13].

There may be concern about antibiotic misuse by CHWs in the community for treatment of pneumonia. However, it has been shown that trained CHWs using standard case management of pneumonia can reduce both pneumonia specific and infant mortality [15,16]. Moreover, standard case management of pneumonia at facility and community level improves rational use by reducing inappropriate antibiotic usage [17–20], potentially resulting in less pressure on antimicrobial resistance. However, in Asian countries majority of care seeking for suspected pneumonia is from private providers and one of the reasons for seeking care from private providers is the increased likelihood of receiving injections for pneumonia and antibiotics for diarrhoea, which is perceived to be an appropriate treatment [13]. Irrational drug use by general physicians and occasionally by pediatricians has been reported from Pakistan as well [21–23].

To extend health services to the community and household level, Government of Pakistan launched the National Programme for Family Planning and Primary Health Care, hereafter called Lady Health Worker (LHW) Programme in 1994. This community based literate woman trained as health worker is known as LHW. She provides health education, family planning and curative care for childhood illnesses including pneumonia. By 2007, over 90 000 LHWs were trained and deployed in rural and semi–urban areas, covering 60% of the population [24]. An independent program evaluation showed that care seeking for children with respiratory infection from LHWs was reportedly only 16% [21].Most families believed such consultation from LHW was unnecessary. The national LHW Programme (LHWP) and Save the Children with technical support from WHO implemented a community mobilization program to improve appropriate care seeking for pneumonia in children in Haripur district, Pakistan. It was part of a cluster randomized clinical trial for treatment of pneumonia for children under five years of age, which has been reported elsewhere [25].

## METHODS

### Study design and objective

This was a cross-sectional study with pre and post intervention assessments for pneumonia care seeking coverage. The objective of the study was to increase the number of children receiving treatment for suspected pneumonia by LHWs through community mobilization approaches for prompt recognition and care seeking in 2 to 59 month–old children.

The primary outcome was the proportionate change in care–seeking from LHWs by caretakers for suspected pneumonia among children 2–59 month-olds. Appropriate care seeking defined as standard case management of pneumonia – National Integrated Management of Childhood Illness guidelines for facility providers and Community Case Management (iCCM) guidelines for community health workers.

### Study setting

Haripur district located in Khyber Pakhtunkhwa Province (KP) is administratively organized into two sub–districts (Tehsils*)* and 44 Union Councils (UC), the smallest administrative unit with a population of 15–25 thousand. In 2008, the projected district population was approximately 856 000, with eighty eight percent living in rural areas [26]. The care seeking interventions were implemented in 28 union councils.

The public sector health infrastructure in Haripur district has one district hospital, five rural health centers (RHCs), 41 basic health units (BHU), 14 other health centers and 750 LHWs. The private sector has seven general hospitals, three maternity homes and several clinics. In addition, local pharmacists and chemists also provide curative care to the community. Each UC has one BHU or an RHC.

LHWs provide preventive and promotive care to children and mothers, and basic curative services for children. LHWs have at least an eighth grade education and receive three months classroom training followed by 12 months of field practice under supervision. LHWs work from health houses established at their residence and serve around 1000 individuals (150–200 families). They conduct five to eight household visits per day, visit each household at least once a month and are available for sick child visits at their health house or the child’s residence. They are linked to the nearest public sector Basic Health Unit (BHU)/Rural Health Center (RHC) for clinical supervision, replenishment of supplies, and in–service training. The LHW is supervised by a lady health supervisor (LHS). Each LHS supervises 15–20 LHWs and visits each LHW at least once a month. The LHS is provided a vehicle, driver and fuel by the LHW programme for field supervision.

The local government structure at district level at the time of the study consisted of elected Nazim (Mayor) who headed the district assembly, which comprised of elected councilors from UCs. Since January 2010, the local government system at the district level is in abeyance and an ad hoc system of administration has been put in place.

### Study interventions

Two community mobilization strategies were implemented in the 28 union councils. The main purpose was to create awareness on signs/symptoms of pneumonia, importance of early care seeking, and the role of LHWs in treatment of pneumonia.

**1. District and union council level sensitization**

Individual and group meetings at district, tehsil and union council levels with district, tehsil and UC Nazims, religious leaders, teachers and male and female councilors were conducted. Senior staff from the project and LHWP conducted group and one to one meetings. The discussions focused on pneumonia burden, signs of pneumonia, importance of early recognition of pneumonia at household level and prompt treatment at community level and the project objective to strengthen LHW capacity to educate families to recognize signs and symptoms; diagnose and treat pneumonia; and refer cases to appropriate health facility, where needed. Formative research findings showed that the main reason for low care seeking from LHWs was community perception that LHWs provide only health education to mothers and children, with limited or no role in treatment of illness. During the meetings clear messages around LHWs’ capacity to manage pneumonia and the additional training she received was emphasized. Support from community leaders was sought for disseminating messages to their respective communities, and motivate households to seek care from the LHW. Individual meetings were followed by formal presentations at district and UC assemblies and UC level sensitization meetings for male and female councilors. In turn, the opinion leaders spoke about the initiative in the relevant forums and their regular interaction with community members. The District Health Board (DHB) headed by the district Nazim was actively engaged and updates on project activities were provided during the DHB quarterly meetings.

**2. Community level sensitization**

**Training of lady health workers, male promoters and development of job aids.** According to LHWP guidelines, in each LHW catchment area, women and male health committees were to be established to engage the community in LHWs’ activities and to create awareness. It required each LHW to conduct one community session per month on maternal and child health issues in her catchment area. However, the pre service training did not prepare her well enough to engage with women in the community. In most cases either the committees were nonexistent or non–functional. As part of this project women and men health committees were reactivated or established, where absent.

As LHWs’ access to male community members was low due to cultural barriers, we requested communities to nominate a male volunteer as health promoter from LHW catchment area to facilitate her work and to conduct community awareness sessions for male community members. The LHW and the male promoter from LHW catchment area were trained to strengthen their skills for community engagement and facilitating community sessions. The training included classroom sessions, role plays and supervised community session. Initial two day training was followed by regular on site mentoring by the LHS and six monthly refreshers. Based on the qualitative research findings, pictorial counseling cards with culturally appropriate messages on signs of pneumonia and appropriate treatment, sources of care and home care for the treated child were developed. The LHWs and male promoters were trained to use the materials to give simple and clear messages to the community. In all 511 LHWs and 511 male health promoters from 28 UCs were trained.

**Community sessions.** After the training LHWs and male health promoters started conducting community awareness sessions (CAS) for female and male community members respectively. Each LHW conducted at least one CAS monthly attended by 8–15 mothers and elder women in the community. Care takers, whose children had been treated by the LHWs also attended the sessions and shared their experiences with other women. These sessions helped create awareness on pneumonia signs, timely and appropriate care seeking from LHWs in their communities. Another objective was for the session participants to share the messages and experiences with their kin and neighbors. The sessions were periodically monitored by the LHS for quality purposes. Also, during her daily visits to 5–7 households, LHW counseled mothers and other female caretakers using counseling charts with pictorial messages. The male activist conducted scheduled periodic sessions and also when requested by the LHW. The methodology and content was same as in the LHW sessions. Fathers whose children had been treated by the LHWs also attended the sessions as advocates for care seeking from LHWs. Examples of community experience are given in **Box 1**.

Box 1 **Community experience**

**Case 1**: Union Council Ali Khan, Village Kaal, District Haripur

Abdul Mannan, Naib Nazim (Deputy Mayor), UC Ali Khan, Haripur, never considered LHW as a health care provider. He thought that LHWs were deployed to provide health education to community women and didn’t know that they could treat pneumonia with oral antibiotics. During one of the Union Council sensitization meetings, the project staff briefed Abdul Mannan and other council members on the pneumonia project. However, he was reluctant to accept that LHW was skilled enough to treat pneumonia.

In January 2009, his 10 month–old son Abdul Samad became ill with cough and fever. As Abdul Mannan was out of town his wife called him by telephone to inform him about the son’s condition. He enquired about the signs and symptoms of his child^'^s illness. He remembered the information from the sensitization meeting and asked his wife to consult the village LHW. The LHW was called and promptly arrived at their residence. She assessed the child and ascertained that he had pneumonia and gave oral amoxicillin as per standard protocol. The child’s condition started improving after receiving four doses of antibiotic and he was symptom free after completing the full course of treatment. Abdul Mannan has now become a strong advocate for LHWs.

**Case 2:** Union Council Mirpur, Village Rara, District Haripur

Aliya lived in a village 15 km from Haripur town. She has two children and her husband is a day laborer. When Aliya’s son developed cough and rapid breathing she became worried as nobody was at home to take the child to a doctor. Aliya had heard about the LHW but thought it would be waste of time to seek care from her as she believed the proper treatment was injections. She consulted her sister–in–law who lived nearby, who told her that the LHW based in the village had treated many cases of pneumonia, and all of them have recovered completely.

Aliya called the LHW, who examined the child and diagnosed pneumonia and gave oral amoxicillin. The child recovered and she was very happy with the timely availability of quality treatment at her doorstep. Now LHW has become her first point of contact for all child related health problems.

The child’s grandmother said later “*With the efforts of the LHW the life of our child was saved. We gained knowledge from the LHW about the care of a child and now we can educate other people. We have learned many tips on how to prevent our kids from pneumonia. We can now identify it and know what to do in case the child has pneumonia. We have treatment facility at our door step.”*

### Data collection

**Household surveys.** To assess baseline knowledge of care–takers on suspected pneumonia, household and care–seeking practices, and sources of care, a pre–intervention household survey was conducted [27]. Using the formula for sample size for comparison of two proportions for cluster randomized trials [28] and assuming 15% refusals and persons not contactable during the survey we needed to interview a total of 6160 households – 220 households each in the 28 union councils (UC). This would allow us to detect a 20% absolute increase in the proportion of children with suspected pneumonia for whom care was sought from a provider.

A two stage sampling strategy was used. At the first stage four villages were selected at random from each of the 28 UCs. At second stage a fixed sample of 220 households were proportionately distributed among the four selected villages in each UC. One hundred and eighty five HHs were additionally included in the sample for anticipated refusals.

We used a structured questionnaire, adapted from a validated set of 2006 Pakistan Demographic and Health Survey questionnaire. In the sampled household a respondent above the age of 18 was interviewed to determine if a child under five years of age had respiratory symptoms in the two weeks preceding the survey. If a child was identified as having respiratory symptom, the caretaker of the child was asked whether or not they had sought treatment and the source of treatment, if any. The standard DHS definition was used to define “suspected pneumonia”, ie, cough with or without fever and difficulty breathing that was due to a problem in the chest.

Using the same baseline survey methodology and data collection tools a post–intervention survey was conducted to assess the impact of community interventions [29].

Data on number of LHWs treating pneumonia is from the community case management of severe pneumonia cluster randomized trial [25].

### Data analysis

Household survey date was double entered in CSPro software (US Census Bureau) and analyzed in SPSS version 15 (IBM SPSS Statistics).

### Formative research

A qualitative study was conducted in September 2007 to inform the results of the quantitative survey and to assess: the current knowledge of mothers and LHWs about childhood pneumonia; the terminologies commonly used for different signs/symptoms of pneumonia; the home management and care–seeking practices outside home; care taker attitude towards LHWs and other services and use of antibiotics and harmful drugs and; factors that limited care seeking from outside home. Focus Group Discussions were conducted with mothers of children under five years of age in five different Union Councils. Findings were used to improve existing LHW training manual and develop information education and communication (IEC) materials for use during advocacy events and community awareness sessions.

## RESULTS

For sensitizing communities and improving care seeking, senior project and LHW program staff organized 40 male and 73 female meetings for district council members, local councilors, religious leaders, teachers and other opinion leaders from the 28 union councils during the project period. Over a 20–month period a total of 2262 males and 3288 female community members attended the meetings.

The 511 LHWs from the 28 union councils initiated community mobilization activities and started case management of pneumonia as part of project activities [22]. The LHWs organized 6132 community awareness sessions with 50 056 women attending these sessions. Similarly, male promoters conducted 523 sessions with 7845 males attending these sessions. In addition to the direct effect, secondary diffusion occurred through men and women attending the sessions.

The pre–intervention survey was conducted in April–May 2007 and the post intervention survey in April–May 2010. Respondents from 6224 and 6345 households were interviewed in the pre–intervention and post–intervention surveys respectively. The households were similar in terms of ethnicity, educational level of the head of the households, and home ownership.

**Figure 1:**
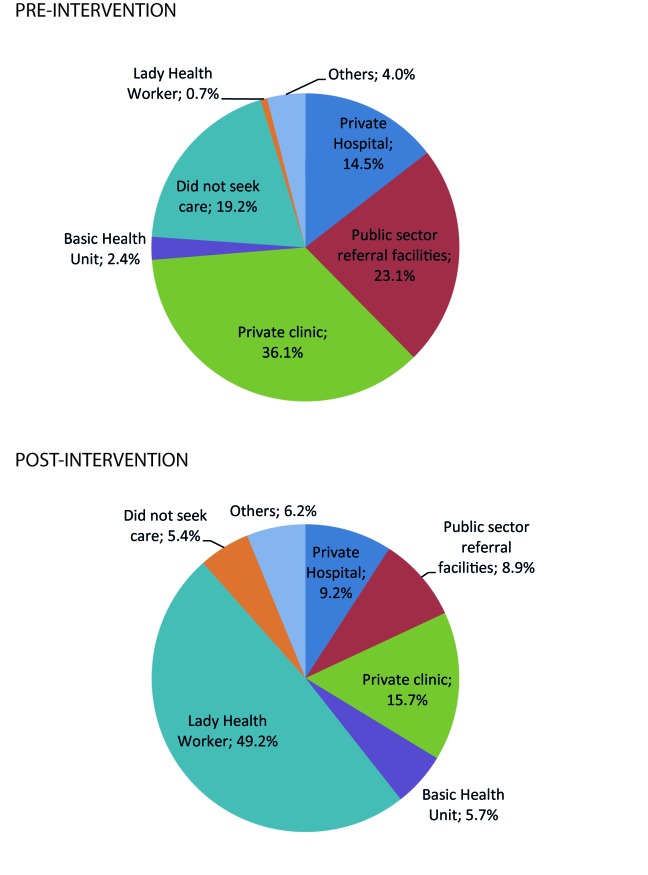
Sources of care seeking for children under five years of age with suspected pneumonia from pre and post intervention household surveys.

In pre–intervention survey, care takers of 80.8 of children under five years of age with signs of respiratory illness in the last two weeks reported seeking care for the child’s illness. Majority (36.1%) of the care takers sought care from private clinics followed by public sector referral facilities (23.1%) and private hospital (14.5%) (**Figure 1**). Only 2.4% sought care from Basic Health Units and 0.7% from the LHWs [24]. The main reason cited for not seeking care from the LHWs was lack of knowledge about their ability to treat pneumonia.

In the post–intervention survey, the overall care seeking for children with cough and difficult or fast breathing in the last two weeks increased to 95.6% [26]. Besides increase in overall care seeking, substantial changes occurred in care seeking patterns. The majority (49.2%%) of care takers sought care from the LHWs, followed by private clinics (15.7%), private hospital (9.0%), and public sector referral facilities (8.9%). The number of children seeking care from BHUs also increased (5.7%).

**Figure 2:**
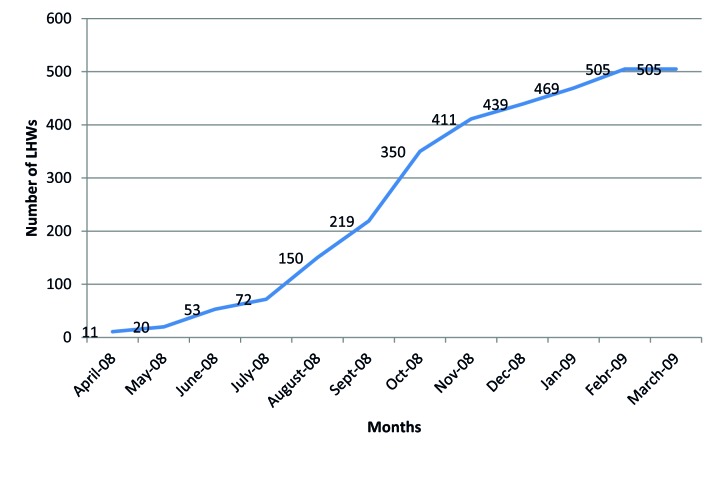
Lady Health Workers (LHWs) who reported treating childhood pneumonia after initiation of project activities in April 2008.

Besides increase in care seeking for pneumonia from LHWs, in one year period the number of LHWs treating children with pneumonia increased from 11 in April 2008 to 505 in March 2009 (**Figure 2**).

We also calculated the proportion of expected cases treated by the LHWs for the April 2008–March 2009 twelve month period. Using estimated incidence of 0.41 episodes per child/y for Pakistan [30] we calculated that of the 31 462 (0.41 × 76735) expected cases of pneumonia in children under five years of age in the 28 union councils, LHWs managed 14 057 cases, ie, 45% of the cases. This validates the post–intervention care seeking figure of 49.2%% from LHWs.

## DISCUSSION

Our results show that community mobilization interventions led to an overall increase in care seeking for pneumonia, particularly from LHWs, which increased from 0.7% to 49.2% in three years. Additionally, the number of LHWs treating pneumonia cases went up from 11 to 505 in twelve months. In the 28 union councils of District Hairpur, LHWs treated 45% of all expected cases of pneumonia.

The increase in care seeking from LHWs resulted in a shift in care seeking from private clinics and hospitals and other referral facilities. Other studies have also observed the effect of community interventions to create awareness on behavior change. An Indian study looking at improving prevention and treatment of malaria through community mobilization also found shift in care seeking to community health workers and increase in prompt diagnosis and treatment [31]. A study implementing programme to create care taker awareness about danger signs through group and one to one health education sessions in peri–urban areas of Lusaka, Zambia increased care seeking for children with danger signs from 56% at baseline to 65.8% at follow–up three years later [32,33]. The study found that educating caretakers on danger signs and need for prompt action through appropriate interventions can change behaviors, overcome distance and cost barriers, and increase care seeking. The shift in care seeking from health facilities to LHWs benefited the community by bringing free and quality care closer to home resulting in reduced burden on the families in terms of costs for travel and treatment [34]. Furthermore, it diminished burden on the already overstretched public sector facilities. The companion treatment trial found that children referred for treatment of pneumonia received non–standard treatment in the shape of multi–drug therapy [22]. This is similar to the inappropriate prescribing practices reported elsewhere from Pakistan [19,20].

It is reported that although the deployment of CHWs brings care closer to the community, it does not always lead to increased utilization [35]. The main reason cited for not seeking care from the CHWs was that families did not know that they existed. This confirms our baseline findings that although the LHWs were working in the community for several years the community was not aware of their ability to treat pneumonia [24]. A community based qualitative study in rural Uganda [36] reported that awareness regarding key pneumonia symptoms and use of antibiotics to treat pneumonia was very low. These findings show that only training, equipping and deploying health providers will not result in appropriate and timely care seeking. A level of trust between the CHW and the community is needed to enable relationships that will produce positive health outcomes [37]. The training of LHWs, provision of supplies and their frequent interaction with communities through CAS has helped create this trust for enhanced care seeking from LHWs by the mothers.

LHW and health promoter training to conduct community awareness sessions using culturally appropriate messages and pictorial health education material helped increase awareness, thus improving care seeking. In addition to the direct effect, we believe secondary diffusion occurred through men and women attending the sessions. Parents of children treated by LHWs proved to be strong advocates. Individual advocacy efforts with elected representatives from District to Union council level, teachers, religious leaders, organization of sensitization meetings at local levels and participation and highlighting the issue of pneumonia in District Health Board also played a substantial role in highlighting LHW role in treatment of pneumonia.

A major strength of the study was the Ministry of Health–Save the Children Public–Private partnership and implementation of the project interventions within the existing LHWP structure. LHWs are mandated to carry out community mobilization activities including conducting education sessions on health promotion, prevention and prompt treatment, where necessary. We mainly streamlined and strengthened this component. Minimal additional inputs were provided during the study in the form of training for sensitization and conducting community awareness sessions and monitoring. Second was the involvement of public representatives and other major stakeholders at district and community level at all stages of programme implementation. They became a major resource for information dissemination and problem solving. Finally, the project overcame resistance encountered from physicians and other local health providers as they feared loss of income with LHWs treating pneumonia cases at community level. We conducted sensitization seminars with local health providers and physicians to explain the project objectives and their cooperation was sought to provide referral support.

A general limitation of the before–after design is the change over time influenced by rapidly improving sources of information for improving knowledge and practices and introduction of interventions by other groups. As CAS were carried out across the board in all 28 UCs with no control group, it is possible that other factors may have played a role in improving care seeking. However, we believe that other factors can be eliminated as contributors to change in care seeking for two main reasons; first, to our knowledge there were no campaigns on print or electronic media or by other organizations to disseminate information on pneumonia prevention and care seeking during the study implementation period to which the community may have been exposed; second, we have been working with the public and private sector in the district for over a decade and know that there were no interventions directed towards LHWs or the community to improve care seeking or service delivery at LHW level. The household survey findings of increased care seeking from LHWs are also validated by the routine LHW program service delivery data. Another limitation of our study was the DHS definition for suspected pneumonia. This is not a robust measure and may have overestimated the two–week prevalence of suspected pneumonia. In addition, despite clear definition for sources of care, care takers may have not correctly reported the sources of care. As our sample size was large and the same set of questionnaires and training methodology was used for both pre–intervention and post–intervention surveys, it would not have affected the results for the two time periods.

## CONCLUSION

The study findings show that it is possible to mobilize communities to improve appropriate care seeking practices at scale within the public sector. The experience showed that while some extra support was provided to increase community mobilization and retrain LHWs, some of the other key elements such as health committees, LHWs and LHSs already existed in the programme. We also showed that it is important to target both direct community awareness activities at village level, and involve stakeholders at district and sub–district level. Applying the lessons learned from the study in other districts of Pakistan will not require additional inputs in terms of money and human resources. However, to sustain momentum of community mobilization interventions in district Haripur and replication in other districts, it is important that senior provincial and district program managers realize the significance and effectiveness of community engagement interventions and ensure its inclusion in annual work plans and budgets.

The study findings have major implications for Pakistan’s LHW programme, where on an average only 16% of children with ARI utilize LHW services for case management [23]. As only a few countries have such a large scale community based programme, the LHW programme provides a huge opportunity for improving access to remote communities and accelerating mortality reduction through standard case management.

We have shown that communities are willing to seek care for quality services, even if they are provided by CHWs. Other large scale community case management programmes in Asia and Africa can benefit from the strategies implemented in Haripur district. Finally, governments and donors making huge investments in infrastructure, capacity building, and medicines have to be cognizant that these inputs are not sufficient to improve treatment services. The experience of LHWP underscores this point. Since its launch 20 years ago and despite having reasonable financial and human resources, it has remained underutilized in the delivery of curative care services. We recommend that in addition to strengthening CHW skills in service delivery, community mobilization and behavior change activities should be integral part of such programmes.
